# Thermographic imaging of police working dogs with bilateral naturally occurring hip osteoarthritis

**DOI:** 10.1186/s13028-020-00558-8

**Published:** 2020-11-10

**Authors:** João Carlos Agostinho Alves, Ana Margarida Moniz Pereira dos Santos, Patrícia Isabel Figueiredo Jorge, Catarina Falcão Trigoso Vieira Branco Lavrador, L. Miguel Carreira

**Affiliations:** 1 Divisão de Medicina Veterinária, Guarda Nacional Republicana (GNR), Rua Presidente Arriaga, Lisbon, 9 1200-771 Portugal; 2 MED – Mediterranean Institute for Agriculture, Environment and Development, Instituto de Investigação e Formação Avançada, Universidade de Évora, Ap. 94, Pólo da Mitra, 7006-554 Portugal; 3 Faculty of Veterinary Medicine, University of Lisbon, (FMV/ULisboa), Lisbon, Portugal; 4 Interdisciplinary Centre for Research in Animal Health (CIISA), University of Lisbon, (FMV/ULisboa), Lisbon, Portugal; 5 Anjos of Assis Veterinary Medicine Centre (CMVAA), Barreiro, Portugal

**Keywords:** Digital thermography, Dog, Hip, Osteoarthritis

## Abstract

**Background:**

Digital thermal imaging is a physiologic, non-invasive, contactless, and non-radiating diagnostic tool that can assess a wide range of musculoskeletal conditions, including hip osteoarthritis (HOA). Fifty police working dogs were evaluated to compare the dorsoventral (DV) and lateral (LT) thermographic images in dogs with naturally occurring bilateral HOA. A DV, and left and right lateral LT images were obtained for each animal in six different moments. They were positioned standing in a symmetrical upright position for the DV view. Each image included the area from the last lumbar to the first coccygeal vertebrae. Each LT view was set with the greater trochanter in the centre of the image. Images were taken with a thermographic camera from a distance of 60 cm. Mean and maximal temperatures were recorded, analyzed with ANOVA, dependent samples t-test, and Spearman correlation, with P < 0.05.

**Results:**

Nine hundred images were considered, collected from 30 males and 20 females, with a mean age of 6.5 ± 2.2 years and bodyweight of 26.7 ± 5.3 kg. The overall value recorded on the DV view was 25.3º ± 9.1 and 28.4º ± 2.8 on the lateral view. These were significantly different (P < 0.01) and with a low correlation (r = 0.10, P = 0.03). German Shepard dogs showed significantly lower values on all views than other breeds (P < 0.01), and heavier dogs had higher values on the lateral view.

**Conclusions:**

This is the first study that describes digital thermography's diagnostic use to evaluate working dogs with naturally occurring HOA, comparing two different views. Future studies should address each one's value in the diagnosis and response to treatment of this disease.

## Background

Hip osteoarthritis (HOA) is a common problem in the canine population. The severity of clinical signs is variable, and many cases are subclinical [1]. Hip dysplasia is a significant predisposing factor, and other factors include age, prior joint injury, obesity, genetic predisposition, and activity levels. Overall, osteoarthritis accounts for at least 80% of cases of lameness and joint diseases in companion animals [2–5].

Digital thermal imaging is a physiologic, non-invasive, contactless, and non-radiating diagnostic tool that relies on heat resulting from physiological functions related to skin temperature control [6–8]. Skin temperature reflects a complex system that depends on blood-flow rate, local structures of subcutaneous tissues, and the sympathetic nervous system's activity. Inflammation in subcutaneous and deeper tissues can be reflected by superficial tissue temperature changes due to changes in blood vessels' diameter, blood flow rate, and increased capillary permeability [9, 10]. Digital thermal imaging can be used to assess a wide range of musculoskeletal conditions, including OA, by identifying tissue’s temperature changes. It is also useful to monitor rehabilitation progress, and, unlike other medical modalities, it is not related to morphology [7, 11, 12]. A 180 × 180 pixel resolution has been deemed enough to provide reliable results for medical uses, but a higher resolution (320 × 240) means smaller changes can be detected [13]. It has been described as useful in several species, from humans to horses and cats, but its clinical utility has rarely been studied in companion animals [7, 13–15]. By correlating changes in temperature patterns with various diseases, degenerative, or injury processes, digital thermography can provide a reproducible diagnostic tool, particularly in early disease phases [16–18]. Canine thermal imaging has been documented recently [11], with a growing interest reflected in an increase in the number of studies assessing thermography use. These reports present this diagnostics modality to evaluate a wide range of pathologies in different joints and the inter-vertebral disc. Type and coat colour are variables that must be taken considered, as its influence on the evaluation results has been documented [12, 13, 19–23].

## Methods

This study aimed to compare a dorsoventral (DV) and a lateral (LT) thermography image views of police working dogs presenting with HOA. We hypothesize that both views present similar results. The study protocol was approved by the ethical review committee of the University of Évora (Órgão Responsável pelo Bem-estar dos Animais da Universidade de Évora, approval nº GD/32,055/2018/P1, September 25th, 2018). Written, informed consent was obtained from the institution responsible for the dogs (Guarda Nacional Republicana, Portuguese Gendarmerie). The sample comprised 50 active police working dogs with naturally occurring bilateral HOA, signaled from the population of police working dogs of the Guarda Nacional Republicana. These animals were submitted to treatment for HOA and to be included in the study, they should have a bodyweight ≥ 15 kg, be over 2 years, and on no medication or nutritional supplements for six weeks or more. The study was conducted over 180 days, with images collected on days 0, 8, 15, 30, 90, and 180. On each day, three images were taken sequentially from each animal: a dorsoventral view, a right lateral view, and a left lateral view. All images were obtained with a FLIR ThermaCAM E25® camera.

**Fig. 1:**
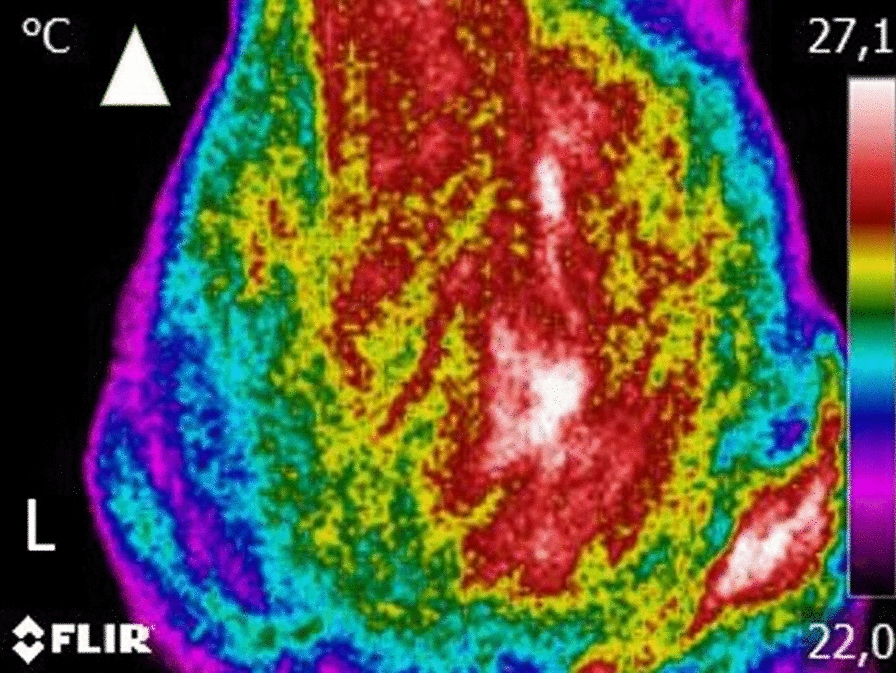
Example of a digital thermography dorsoventral view. Each image included the area from the last lumbar vertebra to the first coccygeal vertebra at a minimum at a distance of 60 cm. Arrowhead indicates cranial direction

Before the images were obtained, dogs were allowed to walk calmly around a room with a steady temperature, set at 21 ºC, to adjust to room temperature for 30 min. For the dorsoventral view, animals were positioned standing in an upright position, as symmetrically as possible. If needed, the trainer could help place the dog by holding the dog under the abdomen but without touching the dog’s torso. Each thermographic image included the area from the last lumbar vertebra to the first coccygeal vertebra at a minimum at a distance of 60 cm (Fig. 1). This procedure has been previously described, with high repeatability between observers and cameras [14]. Each lateral view was set with the greater trochanter in the centre, also at a distance of 60 cm. The range of temperature was set at 15–40°C and emissivity at 0.98.

**Fig. 2:**
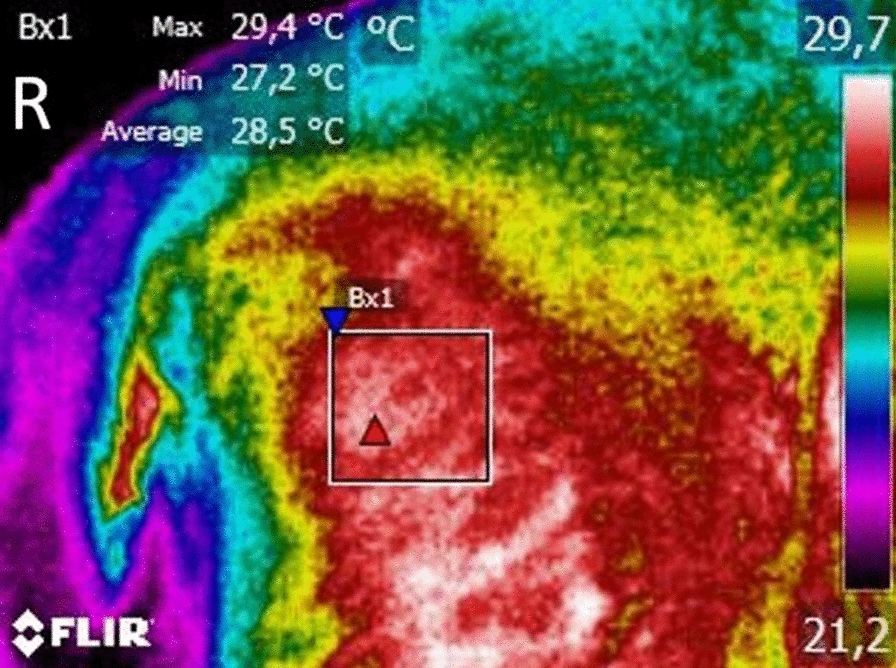
Example of data analysis on a digital thermography lateral view. A temperature box is placed over the anatomical area of the hip joint, and temperature values recorded

Data from the thermographic images was analyzed with the free software Tools (FLIR Systems, Inc), and the Rainbow HC collor pallet was used. Both hips' mean temperatures on the two views were evaluated by placing temperature boxes of equal size on the anatomical area of the hip joint (Fig. 2). The maximal temperature within the box was also registered since the goal was to record signs of inflammation, a hallmark of OA. Normality was assessed with a Shapiro–Wilk test, and mean values obtained on the dorsoventral view were compared with those obtained on the lateral view with ANOVA, followed by an LSD post-hoc test. The same procedure was conducted for maximal temperatures. Temperature values were compared by age, sex, breed, and body weight using a paired samples t-test. For weight, cut-off values of 20, 25, 30, and 35 kg were evaluated. Correlations were assessed with the Pearson correlation coefficient. All results were analyzed with IBM SPSS Statistics version 20, and a significance level of P < 0.05 was set.

## Results

The sample included 50 police working, and four breeds were represented: German Shepherd dogs (GSD, n = 17), Belgian Malinois Shepherd dogs (BM, n = 15), Labrador Retriever (LR, n = 10), and Dutch Shepherd dog (DSD, n = 8). Thirty were males and 20 females, with a mean age of 6.5 ± 2.2 years and bodyweight of 26.7 ± 5.3 kg. Each joint was considered a sample unit and evaluated individually, and classified as mild (n = 70), moderate (20), and severe (10) according to the Orthopedic Foundation for Animals scoring [24]. Three images were obtained from each animal in six different evaluation moments, amounting to 900 images. Mean and maximal values of overall body weight and age, by breed and sex, are presented in Table 1. Mean and maximal values of overall, left, and right pelvic limbs thermography values on the DV and LT views, by breed and sex, are presented in Table 2.

**Table 1:** Mean and maximal values (± standard deviation) of overall body weight and age by breed and sex

	Bodyweight (kg, mean ± SD)	Age (yrs, mean ± SD)
Overall	26.7 ± 5.3	6.5 ± 2.2
German Shepherd dog	29.9 ± 6.4	5.7 ± 1.8
Belgian Malinois Shepherd dog	27.5 ± 4.1	5.3 ± 1.4
Labrador Retriever	24.3 ± 2.5	8.7 ± 2.5
Dutch Shepherd dog	27.5 ± 4.1	5.3 ± 1.4
Male	29.3 ± 5.4	6.2 ± 2.4
Female	23.5 ± 2.8	6.9 ± 2.5

**Table 2:** Mean and maximal (± standard deviation) values of overall, left, and right pelvic limbs thermography values on the DV and LT views by breed and sex

	Dorsoventral view (º, mean ± SD)						Lateral view (º, mean ± SD)					
	Overall	Overall max	Left	Left max	Right	Right max	Overall	Overall max	Left	Left max	Right	Right max
Overall	25.3 ± 9.1	26.3 ± 1.9	24.9 ± 1.9	26.3 ± 1.9	25.6 ± 1.2	26.2 ± 2.0	28.4 ± 2.8	31.9 ± 3.1	28.7 ± 2.9	31.9 ± 3.2	29.0 ± 2.8	32.1 ± 2.9
German Shepherd dog	24.1 ± 1.9	25.3 ± 1.8	24.1 ± 1.8	25.3 ± 1.8	24.0 ± 1.9	25.3 ± 1.8	27.1 ± 2.6	30.8 ± 3.4	26.7 ± 2.5	30.6 ± 3.6	27.4 ± 2.6	31.1 ± 3.1
Belgian Malinois Shepherd dog	26.0 ± 2.0	26.4 ± 1.9	24.8 ± 1.8	26.4 ± 1.9	27.2 ± 2.3	26.4 ± 1.9	29.1 ± 2.6	32.3 ± 3.0	28.9 ± 2.6	32.2 ± 3.0	29.2 ± 2.6	32.4 ± 2.9
Labrador Retriever	25.8 ± 1.7	27.1 ± 1.7	25.8 ± 1.6	27.1 ± 1.6	25.8 ± 1.7	27.1 ± 1.7	30.7 ± 2.4	32.7 ± 2.7	30.6 ± 2.4	32.6 ± 2.7	30.8 ± 2.4	32.9 ± 2.7
Dutch Shepherd dog	25.7 ± 2.1	26.9 ± 2.1	25.7 ± 2.0	26.9 ± 2.1	25.7 ± 2.1	26.9 ± 2.1	29.7 ± 2.4	32.7 ± 2.7	29.7 ± 2.3	32.7 ± 2.7	29.6 ± 2.5	32.6 ± 2.7
Male	24.9 ± 2.1	26.1 ± 2.1	24.8 ± 2.1	26.1 ± 2.1	24.7 ± 2.1	26.1 ± 2.1	28.6 ± 2.9	31.8 ± 3.2	28.4 ± 2.9	31.7 ± 3.3	28.7 ± 2.9	31.9 ± 3.1
Female	25.6 ± 1.9	26.5 ± 1.9	25.1 ± 1.8	26.5 ± 1.8	26.9 ± 1.9	26.5 ± 1.9	29.4 ± 2.7	32.3 ± 3.0	29.3 ± 2.7	32.1 ± 3.1	29.4 ± 2.7	32.4 ± 2.9

Overall mean values observed in the DV and LT views were significantly different (P < 0.01), and so were maximal values (P < 0.01). Mean and maximal values showed low correlations (r = 0.10, P = 0.03 and r = 0.15, P < 0.01, respectively). Comparing mean and maximal temperatures by individual pelvic limb, significant differences were observed on the left hip on the DV and LT views (P < 0.01). The same was true for the right hip (P < 0.01). Still, a correlation was found between left hip mean values on DV and LT views (r = 0.43, P < 0.01), left hip’s maximal values on DV and lateral views (r = 0.16, P < 0.01), and right hip maximal values on DV and LT views (r = 0.14, P = 0.02).

When comparing male and female dogs, significant differences were observed for mean values of the left and right hips on the LT view (P < 0.01 and P = 0.03, respectively). When animals were grouped by body weight, with the cut-off set at 20 kg, significant differences were observed on the left hip maximal value on the DV view (P = 0.04), on the mean and maximal values of the left hip on the LT view (P < 0.01 for both), and on the mean and maximal values of the right hip on the LT view (P < 0.01 and P = 0.03, respectively). Similar results were observed with the cut-off set at 25 kg, 30 kg, and 35 kg, with significant differences observed on the mean and maximal values of the left hip on the LT view (P < 0.01 for both) and on the mean and maximal values of the right hip on the LT view (P < 0.01 for both). Bodyweight showed a correlation with mean and maximal values recorded on the left LT view (r = −0.34, P < 0.01 and r = −0.30, P < 0.01, respectively), and on the right LT view (r =  −0.30, P < 0.01 and r = −0.23, P < 0.01, respectively).

Comparing animals by breed, mean and maximal values for the left hip on the DV view was significantly different for GSD and BM than all other breeds (P < 0.01). No differences were observed between RL and DSD. A similar trend was observed on the LT for mean values, with significant differences observed between all breeds (P < 0.01), except DSD and RL and BM. For maximal values, GSD registered different values compared to all other breeds (P < 0.01). For the right hip on the DV view, no significant differences were observed between breeds. For the maximal value, significant differences were observed between GSD and all other breeds (P < 0.01), and BM and RL (P = 0.02). On the lateral view, GSD's mean values were again significantly different from other breeds (P = 0.01). Differences were also observed between BM and RL (P < 0.01) and RL and DSD (P = 0.03). For maximal values, GSD registered different values compared to all other breeds (P < 0.01).

## Discussion

Digital thermography provides a visual map of the skin temperature distribution. A difference of more than 1 °C between similar areas or tissues is considered significant, and the identification of inflammation, characterized by, among others, an increase in temperature, is a critical step in determining the appropriate treatment [7, 15]. In dogs, thermography has been able to identify animals with dysplastic elbows and cranial cruciate ligament deficiency, compared with sound animals [20, 22]. All animals included in this study had bilateral HOA, and therefore we only evaluated contralateral differences. When comparing values for the hip joint's anatomical region on the DV and LT views, significant differences were observed. The main reason for this difference may be the larger amount of muscle masses included in the LT view area, thus contributing to higher temperature values. Still, the values observed on different views showed some correlation. This effect was also observed when comparing dogs with different cut-off points for bodyweight and males and females (male dogs being heavier), with increasing body weight always accounting for higher temperature levels. These differences were observed on mean and maximal scores.

A thermographic value of 28.47 °C ± 0.45 on an LT view has been described in healthy LR. However, this value was for a region of interest that encompassed the most proximal third of the pelvic limb and not the hip joint area specifically [19]. Similar values (28.4 °C ± 2.8) were found when the overall sample was considered, but higher values in the LR dogs (30.7 °C ± 2.4). This difference may be associated with OA's changes, but further studies should address this hypothesis.

Working and sporting dog owners and trainers are frequently reluctant to clip their dogs, and it has not been proven as necessary for the thermographic evaluation of structures in dogs. Fur clipping can be harmful during a thermographic evaluation since a minimum of 60 min after clipping is required for stable readings to be acquired [19]. Still, the coat's type and color are variables that must be taken into account, and their influence evaluated, as it tends to be predictable [12, 20, 23]. All of the breeds included in this study had short hair, and some had a double coat. The majority of GSD included were sable, BM fawn, LR yellow, and DSD bridle. Comparing thermography evaluation by breed, the most consistent difference was the lower values observed in GSD, even though these were heavier dogs. The reason for this is unclear but may be related to coat characteristics since GSD usually have a very thick undercoat. Some coats can also make it harder to locate the area of interest, mainly when the hair is very long and/or thick [14, 25]. Strong knowledge of the area of interest's anatomical landmarks is paramount to obtain valuable and correct information from the thermographic images [13]. This identification was not particularly problematic in this study but would undoubtedly be more challenging in longhaired or overweight/obese dogs. These variations in coat further stress the importance of guaranteeing an adjustment period in a temperature-controlled room. In horses, it has been suggested that there is no need for such an adjustment or equilibration time before thermographic imaging. This could be because a horse is much bigger and produces more heat than a dog [26], and also, with less variation in coat types. For those reasons, this suggestion probably cannot be applied to dogs. We also chose to register and analyse maximal scores as higher temperature levels could be related to inflammation. For that reason, it may better reflect the events that occur during OA development.

## Conclusions

This is the first study presenting two different digital thermography views of dogs diagnosed with HOA. Significantly higher values were recorded on an LT view. Coat characteristics influenced thermographic evaluations. Further studies are required to determine each view's interest and the influence of different coats in HOA treatment monitoring.

## Data Availability

The datasets used and/or analysed during the current study are available from the corresponding author on reasonable request.
